# What drives farmers to use conservation agriculture? Application of mediated protection motivation theory

**DOI:** 10.3389/fpsyg.2022.991323

**Published:** 2022-12-13

**Authors:** Khadijeh Bazrafkan, Naser Valizadeh, Setareh Khannejad, Nazanin Kianmehr, Masoud Bijani, Dariush Hayati

**Affiliations:** ^1^Department of Agricultural Extension and Education, School of Agriculture, Shiraz University, Shiraz, Iran; ^2^Department of Rural Extension and Development, College of Agriculture, University of Tabriz, Tabriz, Iran; ^3^Department of Agricultural Extension and Education, College of Agriculture, Tarbiat Modares University (TMU), Tehran, Iran

**Keywords:** psychological analysis, soil conservation, water conservation, sustainable agriculture, adoption intention

## Abstract

Conservation agriculture is an approach for managing agricultural ecosystems, improving productivity, increasing benefits and food security, and preserving resources and the environment. The core purpose of this study was to analyze the constructs affecting the intention to use conservation agriculture measures. For this purpose, protection motivation theory was applied as the theoretical framework. Validation of the model was done using a cross-sectional survey among Iranian farmers, and SMART PLS software was used to test the hypotheses. The results showed that the direct effects of the five constructs of perceived severity, perceived vulnerability, response cost, response efficacy, and self-efficacy were significant on the intention to use conservation agriculture measures. In addition, the variables of perceived severity, response cost, and perceived self-efficacy had significant effects on the fear of not using conservation agriculture measures. The results of the bootstrapping analysis indicated that the fear of not using conservation agriculture measures significantly mediated the effects of perceived severity, response cost, and self-efficacy on the intention to use conservation agriculture. The results of the present research help to develop protection motivation theory by defining new relationships between its variables and achieving a deeper understanding of these relationships. The results also can pave the way for social and psychological interventions in the field of adopting the principles of conservation agriculture in agricultural societies. Finally, the results of this research can be used as a decision-making tool and help for users and planners of behavioral changes to better identify the focus points and necessary strategies.

## Introduction

Increasing population and demand for the use of natural resources such as water and soil have posed many challenges to achieving sustainable agricultural and environmental development. Water shortage, soil degradation, air pollution, pollution of surface and groundwater resources, excessive use of agricultural pesticides, lack of proper drainage of agricultural lands, severe tillage operations, etc. are among the major environmental challenges that the agricultural sector of many countries are dealing with. However, disagreements over how to impose restrictions on the use of environmental resources and the lack of an appropriate database of available resources exacerbate these problems (Van dijl et al., 2014).

Conservation agriculture is emerging as a way to address the serious issues that have endangered the life cycle, environment, and humanity. In other words, conservation agriculture is an approach to managing agricultural ecosystems, improving productivity and sustainability, increasing benefits, food security, and conserving resources and the environment (Lugandu, 2012). Conservation agriculture is a pro-environmental agricultural system that aims to make optimal use of resources and inputs in addition to the protection of water and soil resources. Degradation of soil and water resources, declining groundwater resources, decreasing soil quality, drying of agricultural lands, and increasing soil erosion are some of the problems that have led to the use of conservation agriculture as an exit strategy in recent decades (Swaminathan, 2013). Conservation agriculture is a new paradigm to achieve sustainable agricultural production and a major step in the transition to sustainable agriculture (Farooq and Siddique, 2015).

This agricultural system is based on three principles or bases. First, in conservation agriculture, movements of soil turbulence should be minimized and, if possible, non-tillage or low tillage methods should be used. Secondly, in this agricultural system, efforts should be made to preserve soil cover. The use of crop diversity is the third principle of conservation agriculture (Lugandu, 2012). Milder et al. (2011) state that conservation agriculture can enhance soil fertility, improve nutrient recycling, increase yield (farm profitability), and reduce the need for inputs. In addition, the application of environmental methods and measures such as low tillage systems (which is one of the best methods for agricultural sustainability), can slow down the process of land degradation and increase sustainability in agriculture (Manda et al., 2015). Some studies (see Aune et al., 2012) show that crop yields are higher in conservation agriculture compared to conventional/traditional agriculture. Conservation agriculture conditions such as timely cultivation, more efficient use of fertilizer as a nutrient in the vicinity of seeds, and improving water permeability are among the most important reasons for better performance of agricultural products in this system (Aune et al., 2012).

Mutual et al. (2014) state that despite the benefits of conservation agriculture, most farmers, especially small-holder farmers, do not have the desired behavior and attitude toward its adoption. These researchers claim that this reluctance is generally due to its inconsistency with their agricultural culture and tradition; because many farmers are accustomed to using heavy plows for agriculture. According to Thierfelder et al. (2015), farmers’ fears of a significant reduction in crop production, lack of appropriate and accessible inputs, lack of a proper market for products, lack of access to knowledge and information about the conservation agriculture methods, and poor performance of agricultural extension systems are other factors leading to low acceptance of this agricultural system among members of rural and agricultural communities.

The fact that the development of conservation agriculture in low-income countries in Asia and Africa has been slow indicates a missed opportunity. However, it should be mentioned that with the occurrence of climate change and frequent droughts, the development of conservation agriculture is a necessity (ICARDA, 2018). FAO reports in 2018 show that only 4% of the agricultural lands of Asian countries were covered by the conservation agricultural system. The rejection rate of this agricultural system in developing countries such as Iran is higher than developed Asian countries. For example, surveys by the Iranian Ministry of Agriculture in 2020 demonstrate that only 3.5% of Iranian farmers have used conservation agriculture. According to Gholami et al. (2020), constructs like lack of government support, economic and cultural problems, lack of access to equipment, and adherence to old agricultural methods (traditionalism) of the main constraining factors for acceptance and development of conservation agriculture in Iran. To the authors’ best knowledge, not many (Ataei et al., 2019; Savari and Gharechaee, 2020; Ataei et al., 2021) studies have been conducted on the constructs affecting the acceptance of conservation agricultural activities in Iran. Identifying and analyzing mechanisms predicting farmers’ intentions and conservation behaviors can be very useful in developing adoption of conservation agriculture system. In this regard, present study focused on predicting the intention to adopt conservation agriculture using the individual level predictors and protection motivation theory (PMT). There were three main reasons for focusing on the individual-level predictors of intention. First, focusing on the individual factors provides practitioners and practitioners with a solid theoretical bases for developing social campaigns aiming to facilitate behavioral change in domains such as mobility, home energy use, nutrition, water conservation, soil conservation, etc. (Bamberg et al., 2015). Second, a number of evaluation studies (see Abrahamse et al., 2005; Michie et al., 2009) shows that social campaigns based on individual-level psychological theorizing can effectively change the targeted behaviors of individuals. Third, some researchers (see Möser and Bamberg, 2008; Frederiks et al., 2015) emphasize that any change at the macro level requires behavioral changes at the individual and personal levels of people. The main objectives of present study are as follows:

1. Introducing the PMT and its extended version as a suitable theoretical model for motivating conservation agriculture among farmers.
2. Estimating and analyzing the measurement and structural models of the extended PMT.
3. Evaluating the validation of the extended PMT using statistical fit indices.
4. Critically discussing the applications of this theory for motivating farmers’ intention to adopt conservation agriculture at the national and international levels.
5. Proposing some applicable recommendations and concluding remarks for development of conservation agriculture using the extended PMT.

## Theoretical background: Toward extending PMT

The PMT was first developed by Rogers in 1975, based on the Value Expectation Model (Conner and Norman, 2015). This theory was developed to better understand why and how people respond to potential threats to their own health and safety (Clubb and Hinkle, 2015). In 1983, Rogers modified his theory to introduce a more comprehensive model that included adaptive response costs and maladaptive response rewards in the cognitive mediation equation (Conner and Norman, 2015; Westcott et al., 2017). In this theory, it is assumed that adoption of any behavior in the face of existing threats is a direct response that arises from the motivation of the individual to protect him/herself (Rogers and Prentice-Dunn, 1997; Clubb and Hinkle, 2015). The PMT is a social-cognitive model that requires a person to have protective behavior. This theory evaluates the consequences of individuals’ actions using “threat” and “coping” appraisals (Rogers and Prentice-Dunn, 1997). When people perceive a phenomenon as threatening, they feel threatened and that phenomenon becomes a concern for them. The degree of response to these threatening phenomena is generally influenced by the social-psychological characteristics of the person (Salehi, 2021).

According to PMT, five constructs predict an individual’s intention to commit to a protective behavior: perceived severity of threat, perceived vulnerability to threat, perceived efficacy of a preventive behavior, response costs, and self-efficacy in the face of threat (Pourhaje et al., 2016). Intention is conceptually defined as the willingness of individual to do a particular behavior in the near future (Ajzen, 1991). According to Conner and Norman (2015), protection motivation in PMT is typically operationalized as intention to perform the behavior or avoid the health-compromising behavior. In this regard, in this Trudy, we used intention instead of motivation. These five constructs are considered sub-factors for the above-mentioned general constructs, “threat appraisal” and “coping appraisal” (Rainear and Christensen, 2017).

Threat appraisal occurs when a person decides to engage in risky behaviors. This decision depends on the sources of information that the individual has access to (Rogers and Prentice-Dunn, 1997). Information sources explain potential threats, protective choices, and reasons for whether or not a protective behavior is required (Conner and Norman, 2015). The person should evaluate this information based on insights he or she obtains over time about potential threats and protective reactions. This helps the person to understand whether this information can enable him to deal with the threat or not? (Clubb and Hinkle, 2015). Threat appraisal involves the person’s (who is at risk) expectations of the threat and the resulting harm (Rogers and Prentice-Dunn, 1997). In other words, he/she believes that the given danger has many consequences for him/her. Where the vulnerability and severity of a threat is perceived, a person may experience a certain degree of personal threat (Rainear and Christensen, 2017). The threat appraisal in PMT includes two sub-constructs of perceived vulnerability and perceived severity. Perceived vulnerability refers to the extent to which a person (farmer) believes he or she is vulnerable to a hazard. In contrast, perceived severity emphasizes a person’s belief in the seriousness or likelihood of danger (Sadeghian and Dehghani, 2017).

Copping appraisal refers to a person’s perception of the effectiveness of actions. This process consists of three constructs, self-efficacy, response efficacy, and response costs (Savari et al., 2022). Self-efficacy is defined as the individuals’ belief in their ability to perform a protective behavior successfully (Bockarjova and Steg, 2014; Valizadeh et al., 2021). In other words, this construct refers to the people’s personal beliefs or trust in their ability to perform protective behaviors effectively (Neisi et al., 2020; Valizadeh et al., 2022). For example, if the use of conservation farming activities is considered as one of the principles of dealing with the risks posed by traditional agriculture, self-efficacy will refer to the farmer’s level of confidence in “his/her” actions in this area (Savari et al., 2022). Self-efficacy beliefs contribute significantly to explaining people’s feelings and behaviors (Keshavarz and Karami, 2016). In general, self-efficacy refers to the extent to which people believe they can perform certain tasks to accomplish determined goals (Ung et al., 2015). Some researchers, such as Westcott et al. (2017), consider self-efficacy to be the strongest predictor of behavioral goals. Self-efficacy emphasizes the belief that a person can successfully perform protective behavior. It is worth mentioning that self-efficacy is usually refers to the concept of perceived behavioral control. Due to the fact that perceived behavioral control is seen as a continuum with easily executed behaviors at one end and behavioral goals demanding resources, opportunities, and specialized skills at the other, therefore, demanding resources and opportunities including the time, skills, and economic capacity of the individuals should be taken into account in the behavioral studies (Conner and Norman, 2015). In other words, in measuring self-efficacy, the respondents may be asked to indicate to what extent the specific obstacles (related to the time, skills, and economic situation) prevent them from performing the behavior (Boer and Seydel, 1995; Sheeran and Orbell, 1999). Response efficacy, on the other hand, indicates whether or not the farmer agrees that a protective behavior against risk can eliminate the risk (Sadeghian and Dehghani, 2017). Response efficacy is in fact the individual’s attitude toward the effectiveness of protective measures (Janmaimool, 2017). In addition, it is worth mentioning that response costs are a person’s estimate of the costs (such as money, person, time, and effort) of performing a protective behavior (Sadeghian and Dehghani, 2017).

According to Rogers and Prentice-Dunn (1997), protection motivation results from severity, vulnerability, response efficacy, self-efficacy, a negative function of perceptions of the rewards associated with maladaptive responses, and the response costs of the adaptive behavior. However, Conner and Norman (2015) argue that for protection motivation to be elicited, perceptions of severity and vulnerability should outweigh the rewards associated with maladaptive responses. However, due to the fact that the rewards associated with maladaptive responses are rarely considered as “the conceptual distinction between the reward value of a maladaptive behavior and cost of a protective measure may not be clear” (Abraham et al., 1994), most applications of PMT only consider the main effects of perceptions of severity, vulnerability, response efficacy, self-efficacy, and response costs. Therefore, in present study, we did not employ the rewards associated with maladaptive responses as one of the predictors of intention toward application of conservation agriculture practices.

Some researchers (see Tanner et al., 1991; Floyd et al., 2000; Nelson et al., 2011) have argued in recent years that “fear” can mediate the relationship of perceived severity and vulnerability with intention. In such situations, the sense of fear causes people to evaluate the actions they take to deal with the danger. This evaluation of copping measures can generally lead to the adoption of best practices (Nelson et al., 2011). Therefore, in this study, the variable of fear was considered as a mediator of the relationship between perceived severity and vulnerability with the intention to use conservation agricultural measures. According to Keshavarz and Karami (2016) and Rainear and Christensen (2017), perceived severity and vulnerability also directly affect the intention. In this study, we hypothesized that three constructs self-efficacy, response efficacy, and response cost were also activators of fear. In other words, in addition to directly predicting the intention to apply conservation farming measures and technologies, these three variables indirectly (through fear) affect it. These are the hypotheses that are evaluated in the framework of PMT.

**Figure 1:**
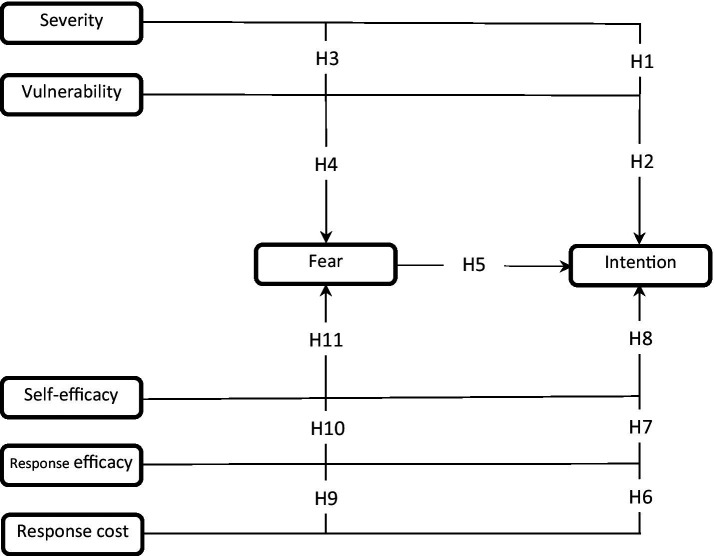
Theoretical model of the study.

In balance, the PMT was configured as Figure 1. Based on this framework, the most important direct hypotheses are as follows:

1. Fear will positively and significantly affect intention.
2. Perceived severity will positively and significantly affect fear.
3. Perceived severity will positively and significantly affect intention.
4. Perceived vulnerability will positively and significantly affect fear.
5. Perceived vulnerability will positively and significantly affect intention.
6. Response cost will negatively and significantly affect fear.
7. Response cost will negatively and significantly affect intention.
8. Response efficacy will negatively and significantly affect fear.
9. Response efficacy will positively and significantly affect intention.
10. Self-efficacy will negatively and significantly affect fear.
11. Self-efficacy will positively and significantly affect intention.

In addition, mediated hypotheses are as follows:

12. Fear positively and significantly mediates the effect of perceived severity on intention.
13. Fear positively and significantly mediates the effect of perceived vulnerability on intention.
14. Fear negatively and significantly mediates the effect of response cost on intention.
15. Fear negatively and significantly mediates the effect of response efficacy on intention.
16. Fear negatively and significantly mediates the effect of self-efficacy on intention.

## Methodology

### Typology of research and study area

From a paradigm perspective, the present research is considered a quantitative study. From the perspective of the applicability of the results, the study is an applied research endeavor. The target area of the study was Fars province in Iran. This province is one of the agricultural hubs in Iran where the agricultural sector and its farmers play a key role in ensuring food security of Iran. Also, the diversity of climate and geographical extent of this province has caused this wide range of agricultural products to be produced in different seasons of the year. It should also be noted that some of the products produced in this province are exported to other countries, and thus contribute to the development of the local and regional economy.

### Population, sample size estimation, and sample selection approach

The target population of this study was farmers in Fars province. According to the Statistical Center of Iran (2019), there are 287,646 farmers in this province. Most of these farmers live in rural areas of Fars province. Sampling was performed using Krejcie and Morgan table (Krejcie and Morgan, 1970). Accordingly, out of 287,646 farmers, 384 were selected for the survey. This sample was the minimum sample size required for the reliability and generalizability of research results to the population. Samples were selected using a multi-step procedure. In the beginning, Fars province was divided into a number of counties based on national geographical divisions, and the number of villages and sub-counties in each county was precisely determined. This paved the way for the use of multi-stage stratified random sampling. Therefore, a sample proportionate to the size was selected from each county. Then this process was done in sub-counties and villages. Finally, each of the samples at the village level was randomly selected and interviewed.

### Data collection tool and measuring the variables of PMT

Data collection was done in 2020. The data collection tool was a three-part questionnaire. The first part of the questionnaire was related to the introduction of the title and purpose of the research. In the second part, the main items measuring each of the variables were presented in the form of a table. This table included four items to measure the response efficacy (efficacy of the use of conservation farming technologies and measures), four items to measure self-efficacy in the use of conservation farming technologies and measures, 20 items to measure the intention to use conservation agriculture technologies and measures, three items for measuring response cost, four items to measure perceived severity, five items to measure the fear, and four items to measure perceived vulnerability (details of the items measuring each of these constructs and their references are provided in Table 1). All the items of these seven constructs were measured using a five-point Likert scale (5: strongly agree to 1: strongly disagree). The third part of the questionnaire included the respondents’ demographic characteristics such as age, level of education, income level, attending training classes related to conservation agriculture, place of residence, and marital status.

**Table 1:** Details of variables and measuring items.

Variable	Items measuring the variables	Sources
Perceived severity of conservation agriculture	Continuous tillage is harmful to soil health.	Tama et al. (2021)
	Excessive use of groundwater resources will lead to a future irrigation water crisis.	
	Excessive tillage by fossil fuel-based tools leads to increased greenhouse gas emissions.	
	The use of chemicals in conventional agriculture increases soil and climate pollution.	
intention to use conservation agriculture measures	Improving soil organic matter	Self-developed
	Using direct cultivation	
	Avoiding continuous tillage	
	Preventing soil from compaction	
	Preservation of vegetation and remnants of previous products	
	Tillage speed optimization	
	Adjust the depth of tillage tool optimization	
	Non-use of very heavy tillage machines and tools	
	Using compound tillage tools (such as combinat)	
	Using Comparison of Fiber Reinforced Polymer (FRP) in chisel plows and composite tillage tools instead of steel blades	
	Replacement of Dulled blades	
	Connecting and adjusting the devices correctly before starting work	
	Using tillage perpendicular to the slope	
	Using modern irrigation methods and optimizing water consumption	
	Wastewater treatment and recycling to the water consumption loop	
	Irrigation during the hours of the day and night when evapotranspiration is minimal	
	Alternative crops with less water consumption	
	Crop rotation	
	Intelligent irrigation management and laser farm leveling	
	Creating small stacks on the farm	
Self-efficacy	Ease of application of conservation agriculture technologies and measures.	Self-developed
	Having the time and skills needed to apply conservation technologies and agricultural practices.	
	Having the necessary economic capacity to apply conservation agriculture technologies and measures.	
	Possessing the knowledge required to apply conservation technologies and measures.	
	Whether or not to use conservation farming technologies and practices is entirely up to me.	
Response efficacy	The use of conservation agriculture technologies and measures are very effective in reducing environmental problems such as water, soil and, air pollution.	Self-developed
	The use of conservation agriculture technologies and measures are very effective in reducing agricultural labor costs.	
	The use of conservation agriculture technologies and measures has a great potential to increase farmers’ profits in the long run.	
	The use of conservation agriculture technologies and measures can help to greatly improve the biodiversity.	
Response cost	The use of conservation agriculture technologies and measures require a lot of effort.	Savari et al. (2022)
	Applying conservation agriculture technologies and measures costs me a lot.	
	The application of conservation agriculture technologies and measures is very time consuming.	
Perceived vulnerability against the risks of traditional agriculture	Traditional agriculture does a lot of damage to the texture and composition of the soil.	Self-developed
	Water resources of the current and future generations have become vulnerable due to traditional agricultural activities.	
	Due to the emission of greenhouse gases from traditional agricultural activities, the health of humans and animals and the environment have been compromised.	
Fear	The decline in income from traditional agricultural activities and its impacts have made rural households economically vulnerable.	Savari et al. (2022)
	I am very afraid of destroying soil resources due to not using conservation agriculture technologies and measures.	
	I am very afraid of the future of surface and groundwater and air pollution due to not using conservation agriculture technologies and measures.	
	I am very afraid of the gradual destruction of the rural and agricultural economy due to not using conservation agriculture technologies and measures.	
	I am very afraid of the destruction of biodiversity due to the lack of use of conservation agriculture technologies and measures.	

### Reliability and validity

To evaluate the overall validity of the latent research variables, a panel of experts reviewed the initial version of the questionnaire. This was necessary to ensure that the items measured what they needed to measure. The evaluation team of the questionnaire consisted of 11 experts in the fields of social sciences, environmental psychology, and agricultural extension and education. Extensive research and educational background in the field of environmental psychology and their field experiences in implementing behavioral change programs in the field of agriculture and environment were used as the selection criteria for these 11 experts. After receiving experts’ opinions about the initial version of the questionnaire, some questions were deleted and/or modified. Also, in some cases, experts suggested new items to measure the constructs. The average variance extracted (AVE) was used to assess convergent validity. According to Wong (2019), constructs whose AVE values are greater than 0.5 have suitable divergent validity. Divergent validity was also assessed using the Fornell and Larcker criterion. According to Hair et al. (2021), if all the values in the diameter of the matrix are greater than the corresponding values in the same column, the researcher can conclude that the Fornell and Larcker Criterion are acceptable. The reliability of the research instrument was evaluated using outer loading factors, Cronbach’s alpha coefficients, rho-A criterion, and composite reliability. Hair et al. (2021), values greater than or equal to 0.7 is considered as the acceptable cut-off values for these indices. VIF values were also applied to evaluate the collinearity among the variables and items. Based on the recommendations of Hair et al. (2021), VIF values should be lower than 3.

### Analysis of the results

Analysis of the results was carried out using partial least square (PLS)-based structural equation modeling (SEM). In this process, three main SEM analyses including measurement models, structural models, and bootstrapping method were employed.

## Results

### Correlations of the constructs

Before running the structural equation model, the correlation between the variables in the conceptual framework (mediated PMT) was investigated using Pearson correlation coefficients (Table 2). The correlation results for mediated PMT’s latent variables showed that perceived severity (*r* = 0.555; *p* < 0.01), perceived vulnerability (*r* = 0.437; *p* < 0.01), response cost (*r* = 0.530; *p* < 0.01), response efficacy (*r* = −0.461; *p* < 0.01), and self-efficacy (*r* = −0.579; *p* < 0.01) have significant correlations with the fear of not using conservation agriculture measures. The significant correlation between these five constructs with the fear of not using conservation agriculture measures indicates the potential role that they can have in explaining the variable of fear and intention. The comparison of the correlation values of these four PMT variables shows that self-efficacy, response efficacy, and perceived severity have the highest correlation values with the fear of not using conservation agriculture measures. Also, perceived severity (*r* = 0.579; *p* < 0.01), perceived vulnerability (*r* = 0.656; *p* < 0.01), response cost (*r* = −0.356; *p* < 0.01), response efficacy (*r* = 0.673; *p* < 0.01), and self-efficacy (*r* = 0.547; *p* < 0.01) have significant correlations with the construct intention to use conservation agriculture measures. Therefore, it can be concluded that these constructs, in addition to fear, have a potential ability to explain the intention to use conservation agriculture measures. The correlation values of response efficacy and perceived vulnerability were higher than the correlation values of other variables with the intention to use conservation agriculture measures. It should be noted that the fear of not using conservation agriculture measures had a positive and significant correlation with intention (*r* = 0.699; *p* < 0.01; Table 2).

**Table 2:** Correlations matrix of the variables.

	Fear	Intention	PS	PV	RC	RE	SE
**Fear**	1						
**Intentions**	0.699^**^	1					
**PS**	0.555^**^	0.579^**^	1				
**PV**	0.437^**^	0.656^**^	0.516^**^	1			
**RC**	−0.530^**^	−0.356^**^	−0.262^**^	−0.164^**^	1		
**RE**	−0.461^**^	0.673^**^	−0.537^**^	−0.391^**^	−0.214^**^	1	
**SE**	−0.579^**^	0.547^**^	−0.600^**^	−0.614^**^	−0.223^**^	0.579^**^	1

Perceived severity (PS), Perceived vulnerability (PV), Response cost (RC), Response efficacy (RE), and Self-efficacy (SE).

** Sig. level: 0.01 error.

### Reflective/measurement models of mediated PMT

At this stage, the reflective/measurement models of the constructs were evaluated (Table 3). Examining these models was necessary for the proper evaluation of the data for structural analysis. The basic reflective/measurement model included seven first-order measurement models. Examining the correlations of the indicators in the measurement models showed that all the items have acceptable loading factors. According to Shyu et al. (2013) and Cheung and Wang (2017), the critical value for accepting factor loadings and maintaining indicators in reflective models is 0.4. In this process, the 7, 8, 10, and 11th items of intention to apply agricultural measures were removed from the model. Because, their factor loadings were lower than the critical value of 0.4. In addition to factor loadings, the weights of each item were also calculated. Based on Hair et al. (2021), the weight of the items actually indicates the relative importance of each of them in the reflective model. This index helps the researcher to identify the items that are less important than the rest of the items and remove them. Eliminating these items helps to make the model fit better. The results of the present study showed that all items in the measurement models had a normal weight distribution. In this regard, none of the items were removed from the model based on this criterion. The evaluation of Cronbach’s alpha, composite reliability, and Dijkstra-Henseler rho A indices for reflective models revealed that the coefficients of all three indices in all variables are higher than the acceptable value of 0.7. According to Hair et al. (2021), values higher than 0.7 for these three indices indicate that the research tool and its constructs are of suitable reliability. The results of AVE index evaluation also demonstrated that the research tool has an acceptable divergent validity. Because, the values of this index for all mediated PMT constructs were higher than the critical value of 0.5. According to Wong (2019), constructs whose AVE values are greater than 0.5 have suitable convergent validity. In addition, the matrix related to the Fornell and Larcker Criterion also indicated that all the values in the diameter of the matrix are greater than the corresponding values in the same column. According to Hair et al. (2021), this result is also one of the criteria confirming the divergent validity of the research tool.

**Table 3:** Evaluation of measurement models and the reliability, validity, and normality of assessment.

**Items/variables**	**Fear**		**Intention**		**PS**		**PV**		**RC**		**RE**		**SE**	
**Loading/weight**	**Loading**	**Weight**	**Loading**	**Weight**	**Loading**	**Weight**	**Loading**	**Weight**	**Loading**	**Weight**	**Loading**	**Weight**	**Loading**	**Weight**
**Fear1**	0.990	0.303												
**Fear2**	0.684	0.309												
**Fear3**	0.541	0.283												
**Fear4**	0.608	0.354												
**Fear5**	0.704	0.343												
**Intention1**			0.784	0.057										
**Intention2**			0.805	0.059										
**Intention3**			0.890	0.067										
**Intention4**			0.953	0.071										
**Intention5**			0.900	0.068										
**Intention6**			0.899	0.068										
**Intention9**			0.956	0.073										
**Intention12**			0.940	0.070										
**Intention13**			0.898	0.069										
**Intention14**			0.954	0.072										
**Intention15**			0.940	0.072										
**Intention16**			0.956	0.072										
**Intention17**			0.938	0.070										
**Intention18**			0.907	0.070										
**Intention19**			0.891	0.066										
**Intention20**			0.940	0.072										
**PS1**					0.964	0.359								
**PS2**					0.672	0.281								
**PS3**					0.606	0.536								
**PS4**					0.673	0.601								
**PV1**							0.696	0.467						
**PV2**							0.768	0.562						
**PV3**							0.659	0.370						
**RC1**									0.812	0.372				
**RC2**									0.760	0.545				
**RC3**									0.714	0.501				
**RE1**											0.623	0.401		
**RE2**											0.672	0.446		
**RE3**											0.699	0.510		
**RE4**											0.901	0.235		
**SE1**													0.907	0.173
**SE2**													0.517	0.159
**SE3**													0.529	0.169
**SE4**													0.607	0.226
**SE5**													0.871	0.693
**CR**	0.88		0.95		0.85		0.853		0.835		0.869		0.88	
**rho-A**	0.833		0.987		0.771		0.812		0.764		0.739		0.845	
**AVE**	0.52		0.829		0.550		0.502		0.580		0.535		0.500	
**Cronbach’s Alpha**	0.74		0.94		0.71		0.77		0.74		0.73		0.76	

Examination of the inner and outer variance inflation (VIF) indices showed that their values for all items and variables were between 1 and 3 (Tables 4, 5). According to Hair et al. (2021), acceptable VIF values are lower than 3. Considering that all these values were less than the critical value of 5, it can be concluded that there is no collinearity between the measuring variables and items. Tables 4, 5 represent the detailed results related to the examination of these indicators.

**Table 4:** Outer VIF values for the measuring items.

**Item**	**VIF value**	**Item**	**VIF value**
**Fear1**	1.137	**Intention20**	2.393
**Fear2**	1.372	**PS1**	1.017
**Fear3**	1.122	**PS2**	1.013
**Fear4**	1.128	**PS3**	1.009
**Fear5**	1.374	**PS4**	1.011
**Intention 1**	2.974	**PV1**	1.114
**Intention2**	2.641	**PV2**	1.111
**Intention3**	2.960	**PV3**	1.148
**Intention4**	2.655	**RC1**	1.093
**Intention5**	2.617	**RC2**	1.111
**Intention6**	2.895	**RC3**	1.093
**Intention9**	2.592	**RE1**	1.084
**Intention12**	2.184	**RE2**	1.097
**Intention13**	2.248	**RE3**	1.070
**Intention14**	2.444	**RE4**	1.033
**Intention15**	2.038	**SE1**	1.343
**Intention16**	2.006	**SE2**	1.377
**Intention17**	2.130	**SE3**	1.497
**Intention18**	2.304	**SE4**	1.584
**Intention19**	2.496	**SE5**	1.134

**Table 5:** Inner VIF values for the measuring items.

	**Fear**	**Intention**
**Fear**		2.212
**Intention**	2.212	
**PS**	1.835	1.933
**PV**	1.698	1.706
**RC**	1.086	1.413
**RE**	1.651	1.664
**SE**	2.227	2.407

### Hypothesis testing using structural/inner model

**Figure 2:**
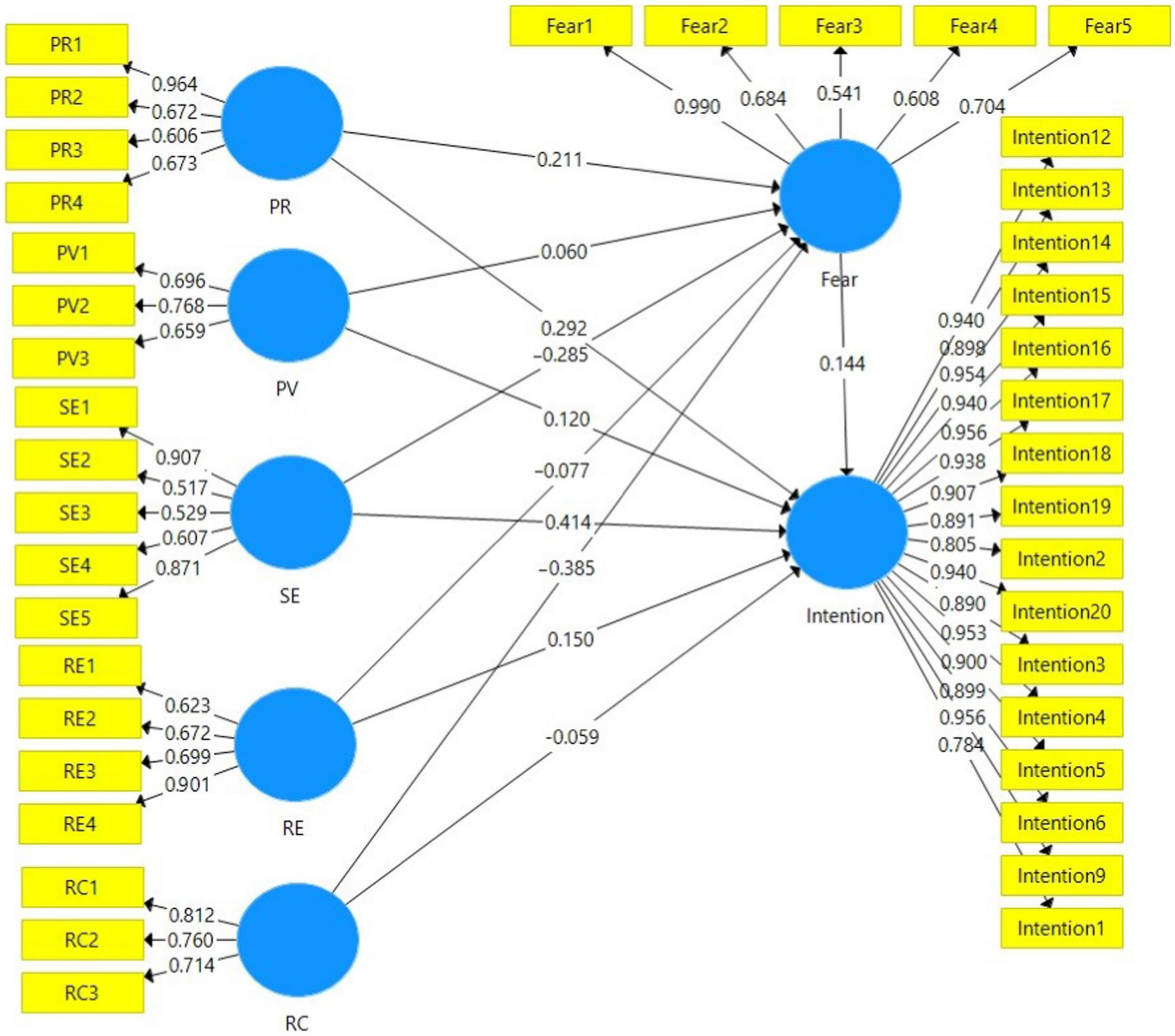
Structural model of mediated PMT.

In this section, the inner or structural model was run using SMART PLS. The implementation of this model was to test the proposed hypotheses. The results of this stage of analysis were presented in Table 6 and Figure 2. The results of the structural model showed that the fear of not using conservation agriculture measures had a positive and significant effect on the intention to use conservation agriculture activities (β = 0.144; *p* < 0.01). This result represents that the first hypothesis proposed in the mediated PMT was supported. In the second and third hypotheses, the effects of the perceived severity on the fear of not using conservation agriculture measures (β = 0.211; *p* < 0.01) and the intention to use conservation agriculture measures (β = 0.292; *p* < 0.01) were investigated. In the fourth hypothesis, the effect of perceived vulnerability on the fear caused by not using conservation agriculture measures was tested. The research results rejected this hypothesis (β = 0.060; n.s.). However, the effect of perceived vulnerability on the intention to use conservation agricultural activities was positive and significant (β = 0.120; *p* < 0.01). In other words, the fifth hypothesis was confirmed by the research results. The results of testing the sixth hypothesis indicated that the response cost has a negative and significant effect on the fear of not using conservation agriculture measures (β = −0.385; *p* < 0.01). Interestingly, the effect of response cost on the intention to use conservation agriculture measures, which was tested in the form of the seventh hypothesis, was negative and significant (β = −0.059; *p* < 0.01). However, the ability of this variable to predict the fear caused by not using conservation agriculture measures was more than the corresponding effect for the intention. Response efficacy was another variable in mediated PMT, which its effects on the variables fear of not using conservation agriculture measures (β = −0.077; n.s.) and the intention to use conservation agriculture measures (β = 0.150; *p* < 0.01) were tested. The results rejected the eighth hypothesis (the effect of response efficacy on fear) and supported the ninth hypothesis (the effect of response efficacy on intention). Testing the effects of self-efficacy on fear caused by not using conservation agriculture measures and the intention to use conservation agriculture measures were the last direct hypotheses of the research. The results demonstrated that self-efficacy has a negative and significant effect on fear (β = −0.285; *p* < 0.01). In other words, the tenth hypothesis was confirmed. However, the effect of self-efficacy on intention was positive and significant, and thus the eleventh hypothesis was also supported (β = 0.444; *p* < 0.01; Table 6).

**Table 6:** Summary of testing hypotheses.

Hypothesis	Path	Beta values	*t* value	*p* value	Result of a hypothesis test
Direct hypotheses					
H1	Fear- > Intention	0.144	4.054	0.001	Supported
H2	PS - > Fear	0.211	3.437	0.001	Supported
H3	PS - > Intention	0.292	7.055	0.001	Supported
H4	PV - > Fear	0.060	1.058	0.290	Rejected
H5	PV - > Intention	0.120	3.818	0.001	Supported
H6	RC - > Fear	−0.385	7.906	0.001	Supported
H7	RC - > Intention	−0.059	2.393	0.017	Supported
H8	RE- > Fear	−0.077	1.315-	0.189	Rejected
H9	RE- > Intention	0.150	2.587	0.001	Supported
H10	SE- > Fear	−0.285	−4.375	0.001	Supported
H11	SE- > Intention	0.414	7.052	0.001	Supported
Indirect (mediation) hypotheses					
H12	PS - > Fear - > Intention	0.030	2.670	0.008	Supported
H13	PV - > Fear - > Intention	0.009	1.044	0.297	Rejected
H14	RC - > Fear - > Intention	−0.055	3.957	0.001	Supported
H15	RE - > Fear - > Intention	−0.011	1.257	0.209	Rejected
H16	SE - > Fear - > Intention	−0.041	3.085	0.002	Supported

After testing the direct hypotheses on the dependent variables of fear and intention, the indirect or mediated (through fear) effects of perceived severity, perceived vulnerability, response cost, response efficacy, and self-efficacy on the intention to use conservation agriculture measures was evaluated. In other words, at this stage, the hypothesis was tested whether the fear can mediate the effect of these five variables on the intention or not. At this stage, the structural model of the research was re-run using the bootstrapping method (Table 6). The results of the bootstrapping analysis indicated that perceived severity has a positive and negative indirect effect on the intention to use conservation agriculture measures (β = 0.030; *p* < 0.01). This result confirmed the twelfth hypothesis of the research. Of course, it should be noted that hypothesis 13, which tested the indirect effect of perceived vulnerability on the intention to use conservation agriculture measures, was not significant (β = 0.009; n.s.). Based on the results of this part of the analysis, the fourteenth hypothesis was significant at the 1 % error level (β = −0.055; *p* < 0.01). In other words, fear significantly mediates the relationship between response cost and intention. The indirect (mediated) effect of self-efficacy on the intention was not significant (β = −0.011; n.s.), which indicates that the research results did not support the 15th hypothesis. Finally, it should be mentioned that the fear caused by not using conservation agriculture measures negatively and significantly mediated the relationship between self-efficacy and intention (β = −0.041; *p* < 0.01). Such a result is the evidence for confirmation of the last/sixteenth hypothesis of the research. In general, the variables in the research framework were able to explain 54.1 and 67.8% of fear and intention changes, respectively (Table 6 and Figure 2).

## Discussion and policy implications

The results of the research indicated that the greater the feeling of fear, the greater the intention of farmers to use conservation agricultural measures. Similar results can be found among the results of Tanner et al. (1991), Floyd et al. (2000), and Nelson et al. (2011). In many cases, farmers do not intend to use conservation agricultural measures because they still do not have a clear idea of the disasters that may occur due to the loss of agricultural resources. This conclusion has been supported by Pradhananga (2014) and Valizadeh et al. (2018) who have investigated the farmers water conservation intention. These researchers conclude that the lack of awareness of the consequences of suboptimal exploitation of agricultural water resources is one of the reasons for farmers’ reluctance to participate in water conservation measures. Naturally, in such a situation, they do not feel worried about the unstable and fragile conditions that they may face in the future. In this regard, it is suggested that the planners and practitioners of agricultural extension increase the fear of farmers regarding the consequences of not using conservation agriculture measures. They can use different strategies for this purpose. Holding discussions with farmers regarding the state of water and soil resources is considered as the first strategy. The second strategy can be focused on broadcasting films or documentaries from regions of Iran or other places that are struggling with various issues due to non-compliance with the principles of conservation agriculture. The third strategy can be focused on the influence of virtual networks. In other words, the planners and practitioners of behavioral change programs in the field of conservation agriculture can improve farmers’ awareness regarding the consequences of not following the principles of conservation agriculture. These three strategies can ultimately increase the fear of not using conservation agriculture measures in farmers, and this can lead to the development of their intention to use the measures.

Based on the results, the greater the perceived severity of traditional agriculture, the greater the intention of farmers to use conservation agriculture measures. This result has been supported by the results of researchers, such as Clubb and Hinkle (2015), Westcott et al. (2017), and Pourhaje et al. (2016). The results demonstrated that perceived vulnerability has a positive and significant effect on the intention to use conservation agriculture measures. In other words, with the increase in farmers’ understanding of the vulnerability of water and soil resources, their intention to use conservation agricultural measures increases. Keshavarz and Karami (2016), Rainear and Christensen (2017), and Nelson et al. (2011) also supported this result in their studies. These results show that many farmers probably still do not understand the severity of the risk related to traditional agriculture and the vulnerability of agricultural natural resources. In this regard, it is suggested to increase perceived severity and vulnerability. One of the most important and short-term strategies for this purpose is to increase perceived severity and vulnerability in farmers through mass media, virtual networks, and part-time training courses. In addition, the second strategy to increase the perceived severity and vulnerability in farmers in focusing on the long-term educational program and social-psychological interventions. The use of this strategy can not only directly improve the intention to use conservation agricultural measures, but also directly lead to an increase in the intention of farmers through the variable of fear.

According to the results, the higher the cost of using conservation agricultural technologies for farmers, the lower their intention to use the technologies. The research results of Keshavarz and Karami (2016), Rainear and Christensen (2017), and Neisi et al. (2020) are in line with this result. It should be mentioned that the response costs are not merely limited to the economic costs. In other words, the cost of response may include temporal, physical, and even intellectual costs. For example, if farmers feel that learning a certain method of conservation agriculture is too time-consuming for them, they will show less intention to use it. Based on result, it is suggested that the policy-makers and planners of conservation agriculture development activities try to use the least expensive methods to introduce this farming system to the farmers. This strategy makes them gradually gain a deeper understanding of the benefits of conservation agriculture and even prepare to accept expensive measures.

From the results of structural equation modeling, it can be concluded that the efficacy of conservation agricultural measures has a key role in improving the intention of farmers. This result has been confirmed by the findings of Karimi et al. (2018), Kim et al. (2022), and Savari et al. (2022). Tangibility of the effects of using conservation agricultural measures is a key feature to strengthen the intention to use this agricultural system. Therefore, it is recommended that special attention be paid to the observability the results of adopting conservation agriculture measures. In other words, it is recommended not to introduce technologies to farmers whose effects can be seen in the long term and gradually. Because, such measures can have a negative effect on the intention of farmers.

Based on the results, the more the farmers consider themselves capable and efficient in conservation agriculture, the greater their intention to use measures. Savari et al. (2022), Pang et al. (2021), and Chen et al. (2020) have reported results similar to this result. The effect of self-efficacy on the intention to use conservation agriculture measures requires psychological interventions of agricultural extension. In this regard, it is recommended that agricultural extension change agents use the verbal persuasion strategy to increase farmers’ self-efficacy in the field of using conservation agriculture measures. In this strategy, farmers receive realistic encouragement in terms of their ability to use conservation agriculture. Through this strategy, they make efforts to use conservation agricultural measures more effectively, and as a result, the probability of obtaining satisfactory results increases.

The results of the research showed that the fear of not using conservation agriculture measures significantly mediated the effects of perceived severity, response cost, and self-efficacy on the intention to use conservation agriculture. It can be concluded that the results of the present study provide evidence of the indirect effect of these variables on the intention. As a result, it is suggested that future researchers employing PMT, consider the mediated effects of fear.

In general, the results showed that mediated PMT has a good ability to predict the intention to use conservation agriculture measures. In other words, this theory can be a reliable theory for creating behavioral changes in agricultural societies. In this regard, it is recommended that officials, policy-makers, planners, and practitioners of behavioral changes in agricultural communities use the mediated PMT and practical suggestions presented in this study to accelerate and improve farmers’ behavioral intentions toward conservation agriculture.

## Conclusion and future research

The general the aim of this study was to analyze the constructs affecting the use of conservation agriculture measures through the lens of PMT. This research ended with the fifth key conclusions. First, the fear of not using conservation agriculture measures significantly mediated the effects of perceived severity, response cost, and self-efficacy on the intention to use conservation agriculture. Second, the direct effects of five exogenous constructs of PMT (perceived severity, perceived vulnerability, response cost, response efficacy, and self-efficacy) on the intention to use conservation agricultural measures were statistically significant. Thirdly, the constructs perceived severity, response cost, and perceived self-efficacy had significant effects on the fear of not using conservation agriculture measures. Fourthly, the theory of PMT mediated by the fear has a high ability to explain farmers’ behavioral intention in the field of conservation agriculture. Fifthly, the results of the current research help to develop this theory and gain a deeper understanding of the relationships between its constructs by defining new relationships between PMT variables. In balance, the general conclusions of present study can pave the way for social and psychological interventions in the field of accepting the principles of conservation agriculture in agricultural communities. In other words, the results of this research can be used as a decision-making tool for the users and planners of behavioral changes to better identify the focus points and necessary strategies.

There were limitations in the current research that should be mentioned to pave the way for future research. First, part of the variance of the intention and fear was not explained in this research. This part of the variance is related to the variables that may be effective in explaining intention and fear, but due to the limitations of the research, they were not investigated in the present study. It is recommended to use more variables to explain intention and fear in future researches. Second, in order to avoid excessive complexity of the structural model, only the mediating effect of the fear was examined. However, future researches can use variables such as place identity and country-mindedness as mediators. Third, in this research, no variable was included as a moderating variable in the model; but, future researches can use variables such as government support as a moderator of the relationship between independent and dependent variables. Fourth, present study employed a self-reporting system for collecting required data, however, future researchers can use other data collection methods for data collection. For example, using qualitative data collection methods can be an effective approach to this end. Fifth, although the results of the present study were conducted with a statistically acceptable sample size and its results can be generalized to other similar areas, future researchers should keep in mind that there is no one-size-fits-all model to conservation agriculture. In other words, mediated PMT may be modified with respect to the context-specific requirements. Sixth, present study is a cross-sectional study with its inherent limitations like being difficult to make a causal inference. Therefore, future researchers are recommended to replicate this study using experimental research designs. Using such studies, they will be able to make causal inference. Seventh, PMT is ultimately used to guide people’s actual behavior. In this research, the actual behavior of farmers was not measured and investigated. It is suggested that future researchers measure the actual behavior of farmers in the field of using conservation agriculture measures and technologies and include it as the main dependent variable in PMT.

## Data availability statement

The original contributions presented in the study are included in the article/supplementary material, further inquiries can be directed to the corresponding author.

## Author contributions

All authors listed have made a substantial, direct, and intellectual contribution to the work and approved it for publication.

## Conflict of interest

The authors declare that the research was conducted in the absence of any commercial or financial relationships that could be construed as a potential conflict of interest.

## Publisher’s note

All claims expressed in this article are solely those of the authors and do not necessarily represent those of their affiliated organizations, or those of the publisher, the editors and the reviewers. Any product that may be evaluated in this article, or claim that may be made by its manufacturer, is not guaranteed or endorsed by the publisher.
